# Characterization and expression of the cytochrome P450 gene family in diamondback moth, *Plutella xylostella* (L.)

**DOI:** 10.1038/srep08952

**Published:** 2015-03-10

**Authors:** Liying Yu, Weiqi Tang, Weiyi He, Xiaoli Ma, Liette Vasseur, Simon W. Baxter, Guang Yang, Shiguo Huang, Fengqin Song, Minsheng You

**Affiliations:** 1 Institute of Applied Ecology, Fujian Agriculture and Forestry University, Fuzhou 350002, China; 2 Faculty of Life Sciences, Fujian Agriculture and Forestry University, Fuzhou 350002, China; 3 Key Laboratory of Integrated Pest Management for Fujian-Taiwan Crops, Ministry of Agriculture, Fuzhou 350002, China; 4 Department of Biological Sciences, Brock University, St. Catharines, Ontario, Canada; 5 School of Biological Sciences, The University of Adelaide, Adelaide, South Australia, Australia

## Abstract

Cytochrome P450 monooxygenases are present in almost all organisms and can play vital roles in hormone regulation, metabolism of xenobiotics and in biosynthesis or inactivation of endogenous compounds. In the present study, a genome-wide approach was used to identify and analyze the P450 gene family of diamondback moth, *Plutella xylostella*, a destructive worldwide pest of cruciferous crops. We identified 85 putative cytochrome P450 genes from the *P. xylostella* genome, including 84 functional genes and 1 pseudogene. These genes were classified into 26 families and 52 subfamilies. A phylogenetic tree constructed with three additional insect species shows extensive gene expansions of *P. xylostella* P450 genes from clans 3 and 4. Gene expression of cytochrome P450s was quantified across multiple developmental stages (egg, larva, pupa and adult) and tissues (head and midgut) using *P. xylostella* strains susceptible or resistant to insecticides chlorpyrifos and fiprinol. Expression of the lepidopteran specific CYP367s predominantly occurred in head tissue suggesting a role in either olfaction or detoxification. CYP340s with abundant transposable elements and relatively high expression in the midgut probably contribute to the detoxification of insecticides or plant toxins in *P. xylostella*. This study will facilitate future functional studies of the *P. xylostella* P450s in detoxification.

The cytochrome P450 monooxygenases (P450s) are a large and complex gene superfamily of heme-thiolate proteins that are found in almost all living organisms^1^. Insect P450s can be divided into four clans: the CYP2, CYP3, CYP4 and mitochondrial P450s, however, the number of genes among insect species can vary considerably (48 in *Apis mellifera* and 164 in *Aedes agepti*^2^)^3^. P450s play many functional roles in insect growth and development, including biosynthesis of hormones as well as inactivation and metabolism of xenobiotic compounds such as pesticides^4^,^5^,^6^.

Mechanisms of insecticide resistance are commonly associated with one or more detoxification genes, including P450s, esterases and glutathione S-transferases^7^,^8^,^9^,^10^. For example, overexpression of CYP6BQ9 in *Tribolium castaneum* brain tissue caused the majority of deltamethrin resistance, possibly through metabolizing the insecticide prior to it binding with the voltage-gated sodium channel receptor^11^ and overexpression of CYP6G1 in *Drosophila melanogaster*, caused resistance to DDT and imidacloprid^12^. Pyrethroid resistance has also been documented through P450 duplications^13^ and through the formation of a chimeric P450 gene, generated by unequal crossing-over between two parental genes^14^.

The diamondback moth (DBM), *Plutella xylostella* (L.) (Lepidoptera: Plutellidae), is a pest of Brassicacea plants with a worldwide distribution. It causes damage to economically important crops, such as canola and cruciferous vegetables, and can undertake seasonal migration and reproduce rapidly (with a life as short as 14 days). DBM has the ability to quickly develop resistance to insecticides^15^,^16^,^17^, and populations have developed resistance to all classes of insecticide, including the bio-insecticide Bt produced by the bacteria *Bacillus* *thurengiensis*^18^.

Several studies have characterized individual DBM P450 genes, including CYP9G2^19^, CYP6BG1^20^ and CYP6BF1^21^, and examined their association with insecticide resistance. Using next generation sequencing, Lin *et al.*^22^ compared differential gene expression between three DBM strains with low, moderate and high levels of resistance to chlorantraniliprole. Compared to a susceptible reference strain, 189 unigenes were overexpressed, 19 of which were P450 metabolic detoxification enzymes. They suggested that these P450 genes play an important role in resistance, as they were differentially expressed in all three resistant strains, while alterations in expression of target site ryanodine receptors were down-regulated in highly resistant strains. Understanding the mechanisms involved in insect resistance development is challenging as the metabolic pathway appears to contain several enzyme subfamilies.

With the rapid development of advanced next generation DNA sequencing technologies, genome-wide investigation of the P450 superfamily in some insect species has improved the identification of the genes responsible for resistance, their expression patterns and biological implications, and our understanding of their phylogenetic evolution^2^,^23^. From these analyses, we have been able to know that *Bombyx mori* has 84 CYP450s that are classified into 26 families and 47 subfamilies^2^. As well, 90 P450s have been identified in *D. melanogaster*^24^, 204 P450s in *Culex quinquefasciatus*^23^ and 112 P450s in *Anopheles gambiae*^2^.

Transcriptomic analysis of DBM previously^25^ identified 137 putative P450 gene fragments, from a set of 84,570 unigenes. In this study, we characterized a complete set of full length DBM P450s sequences (hereafter *Px*P450s) using genomic^26^ and transcriptomic^27^ datasets, and analyzed their phylogenetic relationships with other insects. We also investigated the differential expression of DBM P450 genes in function of the biology at its various life stages and among different strains susceptible or resistant to insecticides. These results provide a basis for further genetic studies on the biological function of P450s in DBM, particularly in relation to insecticide resistance.

## Results and discussion

### Identification and characteristics of PxP450s

A total of 7,180 cytochrome P450 protein sequences from 24 insect species were downloaded from the Cytochrome P450 Homepage^28^ and 18,701 predicted DBM peptide-coding sequences (CDS) were downloaded from DBM-DB^29^. Using the local BLAST and HMMER programs, followed by the FGENESH (+), 85 putative *Px*P450s were identified, of which 84 genes appeared to be functional and one was a probable pseudogene (Table S1). According to the standard nomenclature, the 85 *Px*P450s were divided into 26 families and 52 subfamilies (Table 1). The largest families included CYP340 with 16 genes and CYP6 with 14 genes. Our results support previous estimates by Jouraku *et al.*^25^ who predict that the number of the DBM P450 genes should be between 61 and 137. Interestingly, in the closely related lepidopteran species to DBM, *B. mori*, 84 genes are also found in 26 families and 47 subfamilies^2^.

The average length of *Px*P450s with complete open read frames was 504 amino acids (aa), which is consistent with the average gene length in insects^2^. Multiple-sequence alignments indicated that *Px*P450 proteins had five basic and relatively conserved motifs that were arranged from the N-terminal to C-terminal as follows: helix C, helix I, helix K, PERF and heme-binding motifs (Figure S1). The 85 *Px*P450s were located on 60 scaffolds; 42 of them were found being individually located on a single scaffold, while the rest were on 18 scaffolds with each containing at least two *Px*P450s. A total of 40 genes from 18 clusters were predicted to be tandem duplicates, and they accounted for approximately 47% of all 85 *Px*P450s (Figure S2). Scaffold 130 had the largest *Px*P450 cluster with 4 genes, which belong to the CYP340 family. Similarly, the largest CYP340 family cluster in *B. mori* is also located on a single chromosome^2^. P450 gene families evolved through duplication and diversification^30^, and it has been suggested that clusters of the P450 genes, in species such as *Tetrahymena thermophile*, arose through localized tandem duplications^31^. Complete chromosomal scaffolding of the DBM genome will ultimately enable analysis of *Px*P450 tandem duplication events, providing insights into the evolution and diversification of this gene family.

### Phylogenetic analyses

**Figure 1:**
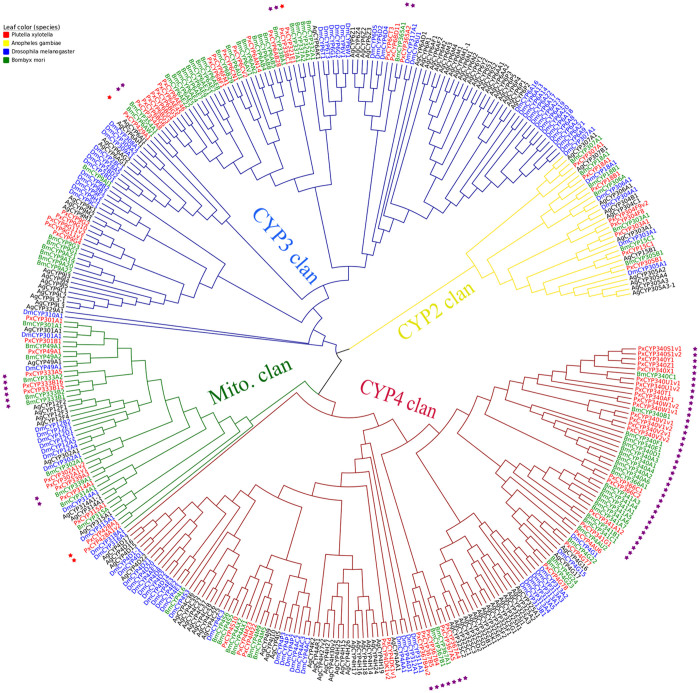
Phylogenetic relationship of the P450s of *P. xylostella* and three other insect species. The phylogenetic tree was divided into four P450 clans, each represented by a branch color. Red, green, blue and yellow branches represent clans of the CYP4, mitochondrial, CYP3 and CYP4, respectively. The four leaf colors are used to distinguish the four species: red for *P. xylostella*, yellow for *A. gambiae*, blue for *D. melanogaste*r and green for *B. mori*. P450s in the four species of insects are named as DmCYP, BmCYP, PxCYP and AgCYP, with the first two letters representing the acronym of their scientific names. The outermost stars in purple show the P450s that are unique in Lepidoptera (*B. mori* and *P. xylostella*), while those in red are DBM-specific.

A neighbor-joining phylogeny of the P450 superfamily was constructed using *P. xylostella* and three other insect species (*A. gambiae, D. melanogaster* and *B. mori*) to identify gene orthologs (Figure 1). The phylogenetic tree resolved the four expected P450 groups including the CYP2, CYP3, CYP4 and mitochondrial clans.

The CYP2 clan contained a single clade with fewer genes and less evolutionary differentiation than the other clans. The branching showed that the two lepidopterans, *B. mori* and *P. xylostella*, were most similar and evolutionally separated from *A. gambiae* and *D. melanogaster. Px*P450s from the CYP2 clan were largely orthologous to *B.mori* (Figure 1), including CYP307A1, CYP18A1, CYP18B1, CYP303A1, CYP15C1 and CYP305B1. The major difference between *B. mori* and *P. xylostella* in CYP2 clan was possible loss of the CYP304F paralogues or orthologues in the silkworm. The genes of CYP2 clan have generally been shown to be evolutionally conserved and play basic physiological functions in insects^31^. For example, CYP303A1 had a single ortholog in all four species. In *D. melanogaster*, it is expressed in the sensory bristles, and plays an essential role in the development and structure of external sensory organs in response to vital mechanosensory and chemosensory stimuli^32^. In *D. melanogaster*, CYP307A1 encodes the Halloween family gene spook, which is required for the biosynthesis of 20-Hydroxyecdysone^33^, CYP18A1 is tightly correlated with ecdysteroid synthesis^34^ and CYP15A1 is involved in juvenile hormone metabolic pathways^5^. As P450 gene clusters commonly have high levels of sequence identity and similar biological functions, the paralogous CYP2 genes in DBM may play similar roles.

In the mitochondrial clan, each species had a similar number of genes, and as expected, *Px*P450s were closely related to the lepidopteran *B. mori*, namely CYP301A1, CYP49A1, CYP333s, CYP302s, CYP314A1 and CYP315A1. Two basal *Px*P450 genes, CYP428As (97% identity), were highly divergent compared with other mitochondrial P450s. CYP333s and CYP339s formed Lepidoptera-specific clusters (Figure 1). Ai *et al*.^2^ proposed that CYP333s are probably related to xenobiotic metabolism. Two single-copy orthologs (1:1:1:1) were also found in this clan: CYP301A1 and CYP315A1. CYP301a1 is involved in the formation of the adult cuticle and ecdysone regulation in *D. melanogaster*^35^. Previous studies^3^ have proposed that two types of mitochondrial P450s are present in insects: genes essential for physiological functions, such as the Halloween genes CYP302A1, CYP315A1 and CYP314A1 that produce the cyclic ecdysteroid pulses triggering moulting^36^ and genes that have rapidly evolved taxon-specific paralogous P450s.

Genes in the CYP3 clan showed multiple expansions in DBM, resulting in species-specific clusters. New functions may have been gained in response to environmental changes^37^. Members of the CYP3 clan are involved in xenobiotic metabolism and insecticide resistance, and some are inducible by phenobarbital, pesticides and natural products^3^. Most *Px*P450s in the CYP3 clan had gene orthologs in *B. mori*, or gene expansions with close phylogenetic relationships. Two main clusters were formed: the CYP6s and CYP9s (Figure 1). In addition, the architecture of introns and exons showed the CYP6s and CYP9s clusters are relatively conserved with two exons split by one intron of phase 1 in the CYP6 subfamily, and 2-1-1-0-1-1-1-0-1 in CYP9 subfamilies (Figure S3). Such conserved gene architectures might result from a recent duplication of the genes and provide functions in response to environmental variations^38^. CYP6BGs, CYP6AEs and CYP9Gs formed small clusters in *P. xylostella*. Previous studies suggest that CYP6s are associated with insecticide resistance. CYP6Bs (CYP6B3, CYP6B4, CYP6B5) can promote the adaptation of *Papilio polyxenes* towards toxin-containing host plants^16^, and CYP6AB11 from *Amyelois transitella* efficiently metabolize several pesticides^8^. Although CYP9G2^19^ and CYP6BF1^21^ have been cloned in *P. xylostella*, their biological roles remain unknown. In contrast, overexpression of *Px*CYP6BG1^20^ has been shown to play a role in resistance to the pyrethroid insecticide permethrin.

Gene expansions were also common within the CYP4 clan, particularly the cluster of CYP340s (Figure 1). Most *Px*P450s in this clan were also clustered with *B. mori*, including CYP4Ms, CYP367Bs, CYP4Gs, CYP341s and CYP340s. The *Px*CYP4DKs were phylogenetically separated from the other clusters, which seemed to have evolved at a relatively early stage. Members of the CYP4 clan are known to encode for constitutive and inducible isozymes linked to odorant and pheromone metabolism and are inducible metabolizers of xenobiotics^3^,^39^. CYP4M1 of *M. sexta* is induced by phenobarbital in the fat body and midguts, and CYP4M1 and CYP4M3 are induced by dietary clofibrate in the midguts^40^. CYP4G61 is thought to be a strong candidate for enhancing the pyriproxyfen resistance of the greenhouse whitefly, *Trialeurodes vaporariorum*^41^. CYP341A2 is preferentially expressed in the chemosensory organs and relates to the chemosensory reception for host plant recognition^42^.

The general clustered *Px*P450 and *Bm*P450 genes indicated their evolutionally close origins^26^ and the possibility that P450s might be quite evolutionally conserved within Lepidoptera. The larger number of CYP3 and CYP4 genes in insects might suggest that they probably play broader functions required for their development or to deal with environmental stress (including exposure to insecticides and plant chemical defenses). Further studies would be required to test this hypothesis.

### Stage- and tissue-specific expression profiles of the PxP450s

**Figure 2:**
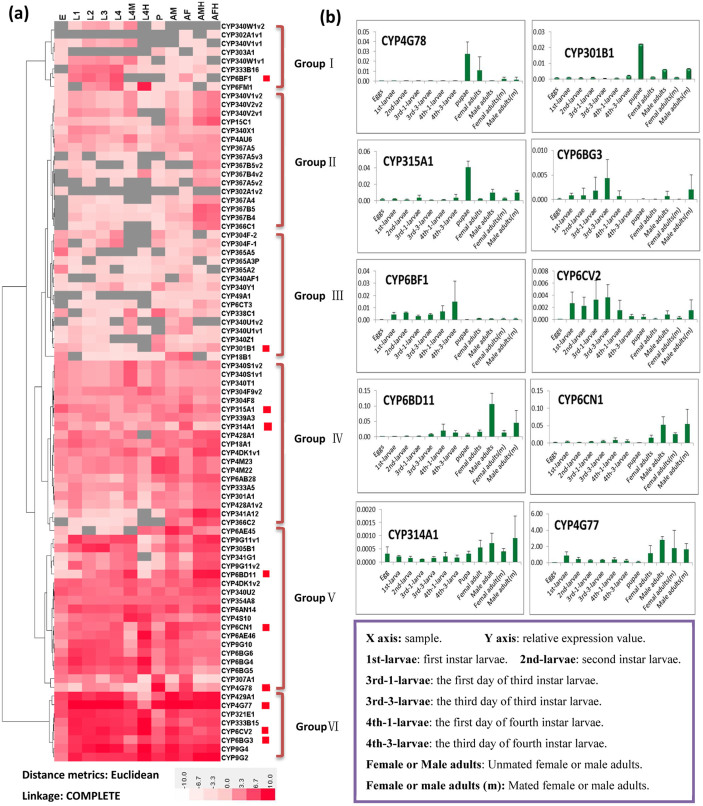
Stage- and tissue-specific expression of the *P. xylostella* P450s. (a) Hierarchical clustering of the 85 *Px*P450s based on RPKM values from the digital gene expression profiling (DGE). The samples are described as in Figure 2a. The log2 RPKM values are presented with varying colors. The darker red represents higher expression values, and the gray in the heat map missing values. (b) qRT-PCR-based expression of 10 selected *P. xylostella* P450s across developmental stages of the susceptible strain.

Expression profiles of 85 PxP450 genes were generated using transcriptome sequence data from multiple developmental stages of the susceptible reference strain (SS) including egg, larva, pupa and adult. Hierarchical clustering across different developmental stages was used to divide these genes into six distinct groups (Figure 2a). Group I consisted of 8 genes that showed higher expression in larval stages than any other stages. Foraging can be essential for larvae^43^, and expression of many diverse detoxification genes may help defend themselves from toxic chemicals produced by host plants. Nearly all of the genes in Group II had higher expression levels in heads than in other tissues or stages. Group III genes exhibited relatively high expression values in eggs and pupae. Especially, the P450 pesudogene, CYP365A3P, was expressed in almost all stags and tissues and have similar expression pattern compared to other two CYP365As (CYP365A2 and CYP365, Figure 2a). Transcribed pesudogenes are contributors to the transcriptional landscape and positioned to play potential roles in regulating cognate wild-type gene expression or function^44^. Group IV showed differential expression, with most genes highly expressed in midguts or heads. Midguts serve as the first line in the detoxification of xenobiotics, which are associated with monooxygenase activities^45^. For example, CYP340S1v1 and CYP340S1v2 were highly expressed in the midguts, and CYP428A1 and CYP18A1 showed higher expression in female and male heads. Group VI contained 18 genes that were expressed at relatively low levels in eggs compared to other developmental stages. Group VI consisted of the remaining 8 genes that had the highest average expression level. qRT-PCR validation of 10 genes with higher RPKM values, which mostly were consistent with the transcriptome sequence data, showed three preferential expression patterns: 1) CYP4G78, CYP301B1 and CYP315A1 in pupae, 2) CYP6BF1, CYP6CV2 and CYP6BG3 in larvae, and 3) CYP6BD11, CYP6CN1, CYP314A1 and CYP4G77 in adults, especially in males (Figure 2b, Figure S4). CYP312A1 in *D. melanogaster* has been shown to be expressed 82-times higher in adult males than in females, and is suggested to be involved in endogenous catalytic reactions^46^. Our results confirm that gene expression of P450s varies with developmental stages and sexes^47^. Although we have inferred some biological functions of the *Px*P450s based on gene expression and homology, the exact functional mechanisms remain to be elucidated.

### Strain-specific expression profiles of PxP450s

To investigate the expression profiles of *Px*P450 genes in three DBM strains, we analyzed gene expression pattern from the 3^rd^ instar larvae using transcriptome data from the insecticide-susceptible genome reference strain, Fuzhou-S (SS), and two insecticide-resistant strains FR (fipronil-resistant, 273.5 fold) and CR (chlorpyrifos-resistant, 3417.2 fold).

**Figure 3:**
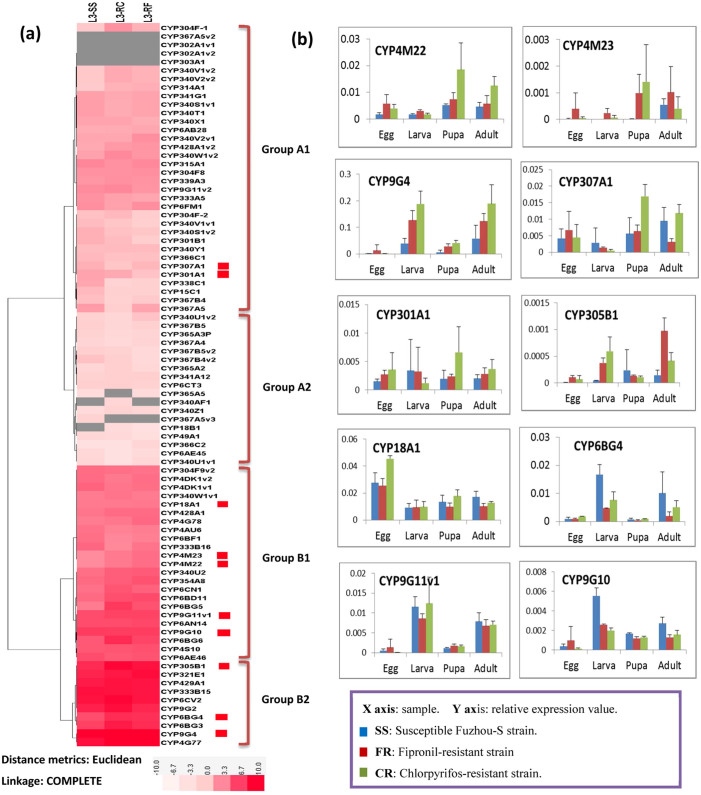
Strain-specific expression of the *P. xylostella* P450s. (a) Hierarchical clustering of the 85 *Px*P450s based on RPKM values from the transcriptome sequencing. The log2 RPKM values are presented with varying colors. The darker red represents higher expression values, and the gray in the heat map missing values. L3-SS: 3^rd^-instar larva from the susceptible strain; L3-FR: 3^rd^-instar larva from the fipronil-resistant strain; and L3-CR: 3^rd^-instar larva from the chlorpyrifos-resistant strain. (b) qRT-PCR-based expression of 10 selected *P. xylostella* P450s across four development stages in three strains.

Based on the heatmap representing the expression profiles of the 85 *Px*P450s genes in these three strains (Figure 3a), two major groups could be defined: Group A had 33 genes with low expression (RPKM < 10.3, average RPKM = 2.3) in the 3^rd^ instar larvae and Group B consisted of 52 highly expressed genes (RPKM > 8.6, average RPKM = 208.6). Each major group could be further classified into two subgroups. Subgroup A1 contained relatively more P450s members showed higher expression values (more than twice) in one or both insecticide-resistant strains relative to the susceptible strain, including CYP304F-1, CYP340V1v2, CYP340V2v2, CYP314A1, CYP340V2v1, CYP428A1v2 and CYP340W1v2 (Figure 3). Over expression of P450 genes commonly causes insecticide resistant phenotypes^23^. Subgroup A2 genes showed no expression differences between the three strains, suggesting little involvement in resistance to these two chemicals. Subgroup B1 genes were highly expressed with some genes, including CYP6BG5 and CYP6BG6 that were overexpressed in the insecticide-resistant strains. Quantitative RT-PCR was used to assess expression of 10 genes with higher RPKM values and variation between resistant and susceptible larvae. Multiple tissues sources were used including egg, whole larvae, (3^rd^ instar), pupae (day 2) and adult (male and female). CYP4M22 and CYP4M23 had higher expression levels in insecticide-resistant strains than in the susceptible strain, supporting the RNA-seq dataset (Figure 3b, Figure S5). The *Px*P450 genes of subgroup B2 showed relatively high levels of expression in all strains. qRT-PCR results indicated that CYP9G4 was up-regulated in insecticide-resistance strains, while CYP6BG4 down-regulated. The expressions of P450s are induced or inhibited by many xenobiotics and some endogenous compounds^48^. Decreased P450 expression is a homeostatic or adaptive response, whereby genes are suppressed in response to pathophysiological signals and various exogenous or endogenous compounds^47^,^49^.

### Lepidoptera-specific P450s

**Figure 4:**
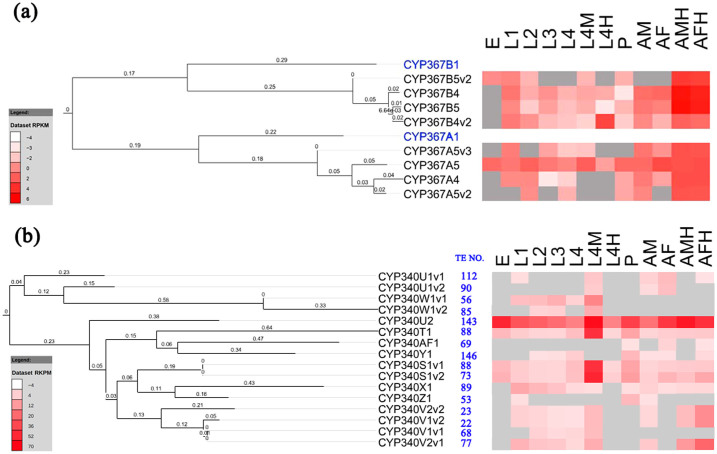
Characterization of lepidoptera-specific P450s (CYP367s and CYP340s). (a) Phylogenetic tree of CYP367s of *B. mori* (in blue) and *P. xylostella* (in black) (left), and stage- and tissue-specific DGE profiles of *P. xylostella* (right). (b) Phylogenetic relationships of the *P. xylostella* CYP340s (left), number of transposable elements for each corresponding gene (middle), and stage- and tissue-specific DGE profiles of *P. xylostella* (right). E: egg; L1: 1^st^-instar larva; L2: 2^nd^-instar larva; L3: 3^rd^-instar larva; L4: 4^th^-instar larva; L4M: midgut of the 4^th^-instar larva; L4H: head of the 4^th^-instar larva; P: pupa; AM: male adult; AF: female adult; AMH: head of male adult; and AFH: head of female adult. The RPKM values are presented with varying colors. The darker red represents higher expression values, and the gray in the heat map missing values.

Multiple P450 clusters were identified that were specific to the Lepidoptera analyzed here, including the clan 4 CYP367s and CYP340s. A phylogeny of the CYP367s (Figure 4a) showed expansions of CYP367A and CYP367B in DBM, compared only one member in these subfamilies in *B. mori*^2^, suggesting that *P. xylostella* may have evolved some specific functions. More interestingly, all *Px*CYP367s showed adult head-specific expression, and CYP367B4v2 tended to specifically express in heads of the 4^th^-instar larva and adult (Figure 4a). P450s specifically expressed in heads probably have specific functions, which is evidenced by fact that CYP6BQ9 predominantly expressed in *T. castaneum* brains confers deltamethrin resistance^11^. In insects, some P450s in the antennae play a role in xenobiotic metabolism or odorant processing, and the antenna is a key site for P450s mediating the metabolism of many exogenous and endogenous molecules^50^. Considering the facts that *Px*CYP367s were greatly expanded and mainly expressed in heads, we suggest that they may play special roles in detoxification or metabolic processing of environmental chemicals.

*Px*CYP340s constituted the largest family of genes in DBM with 16 genes and the largest number of transposable elements per gene (Table 1). CYP340Y1 and CYP340U2 have the most abundant transposable elements inside and in their 2-kb upstream regions (Figure 4b). The phylogenetic tree showed only two clusters, confirming their relative conserved gene intron and exon organization (Figure4b, Figure S3). P450s with abundant transposable elements have been proposed to be involved in induced xenobiotic detoxification^26^. Nearly all *Px*CYP367s showed larva midgut-specific expression. (Figure 4b). In midguts of *M. sexta*, CYP4M1 and CYP4M3 are induced by dietary clofibrate^40^. Midguts serving as the first line in the detoxification of xenobiotics are associated with monooxygenase activities^45^. The CYP340 family with abundant transposable elements and midgut- specific expression might contribute to P450-mediated detoxification in *P. xylostella*.

## Methods

### Identification and nomenclature of the PxP450s

Two approaches were used to identify the putative *Px*P450s. The first method consisted at downloading from the Cytochrome P450 Homepage^28^ (http://drnelson.uthsc.edu/CytochromeP450.html) known P450 amino acid sequences of 24 species and then, subjected the protein sequences of DBM OGSv1 (Office Gene set version 1) (http://iae.fafu.edu.cn/DBM/) to BLASTP analysis with a threshold of P < 10^−7^. The targeted DBM genes that were similar to unknown proteins were verified using BLASTP to search against the NCBI and UniPort databases, and those with the best hit to the annotated P450s were considered to be possible P450s.

The second method used to identify P450 genes was carried out using the HMMER3 (http://hmmer.janelia.org/) software with the default parameters. The P450 domain (PF00067) was searched against the DBM OGSv1 using the hmmsearch program of HMMER3. Then, DBM proteins harboring at least one P450 domain were searched against the Pfam-A dataset that was downloaded from PFAM (http://pfam.janelia.org/) using the hmmscan program and those best-matched domain of P450 were considered as the putative P450s.

We used the online FGENESH and FGENESH+ (http://linux1.softberry.com/berry.phtml) programs to find the entire length of the P450 genes that had no complete open read frames and predicted gene structure based on their whole-length sequences and most similar protein sequences. All the putative *Px*P450s were named by David R. Nelson to maintain consistency in the nomenclature.

### Sequence alignment and motif visualization

The average length of cytochrome P450 genes was approximately 500 aa. Five conserved motifs exist in insect P450s: the helix C motif (WxxxR), the helix I motif (Gx[ED]T[TS]), the helix K motif (ExLR), the PERF motif (PxxFxP[ED)RE) and the heme-binding motif (PFxxGxRxCx[GA])^2^. The fifth conserved motif can be used to easily recognize P450 family members. To visualize the conserved motifs, 77 functional *Px*P450s with more than 300 aa were aligned by ClustalX 2.0.11 using the default parameters, and the five conserved motifs were illustrated using WebLogo^51^ (http://weblogo.threeplusone.com/create.cgi) based on the sequence alignment results.

### Determination of gene location and duplication

*Px*P450s were mapped on scaffolds using Mapchart^52^, and only scaffolds with at least two genes were displayed. Gene location information from the DBM genome (assembly version 2.0) was obtained from the DBM-DB (http://iae.fafu.edu.cn/DBM/)^29^, and genes separated by 0~5 genes were considered to be tandem duplicates^53^.

### Generation of gene architecture and phylogenetic trees

Using the information from the published DBM genome (assembly version 2.0), a figure of the gene architecture was generated online (GSDS) (http://gsds.cbi.pku.edu.cn/). Phylogenetic trees were constructed using the MEGA5.10 based on the neighbor-joining method, including trees of 77 putative *Px*P450s (amino acid residues >300 aa) and four insects (*P. xylostalla*, *A. gambiae, D. melanogaster* and *B. mori*), CYP367s and CYP340s (regardless of the length of amino acid residues). Bootstrap analysis was performed using 1000 replicates, and the Newick output format was used to visualize the phylogenetic tree online with the EvolView^54^ tool (http://www.evolgenius.info/evolview.html#login) or iTOL^55^ (http://itol.embl.de/).

### Expression profile and quantitative RT-PCR analysis

Expression profiling of insect strains, developmental stages and tissue types was generated by Cluster and Treeview software based on the DBM RNA-seq and DGE data, respectively. The RPKM values were log transformed, and genes were clustered in terms of their expression patterns using the similarity metric of Euclidean distance and clustering method of complete linkage.

The susceptible strain and two insecticide (chlorpyrifos and fipronil)-resistant strains of DBM (were reared with radish seedlings at 25 ± 2°C. The median lethal concentrations (LC_50_) of chlorpyrifos- and fipronil-resistant strains were 574-fold (51,500.00 mg·L^−1^ vs. 89.79 mg·L^−1^) and 72-fold (16.85 mg·L^−1^ vs. 0.23 mg·L^−1^) higher than susceptible strain, respectively. Strain- and stage-specific DBM samples were collected and held at −20°C for RNA isolation and qRT-PCR analyses. The strain-specific samples included newly laid eggs, 3^rd^-instar larvae, pupae, and male or female adults of the three strains, while stage-specific samples involved eggs, 1^st^–4^th^ instar larvae, pupae, and unmated or mated males and females of the susceptible strain. Total RNA was extracted from the samples using Trizol reagent according to the manufacturer's instructions (Invitrogen, USA). First-strand cDNAs were synthesized from the total RNA in a 20-μl reaction using the GoScript Reverse Transcription System following the manufacturer's instructions (Promega, USA). Each cDNA sample was diluted to 200 μl for qPCR. Gene-specific primers of 20 *Px*P450 genes were designed using the Primer premier 5 software (Table S2), and the RIBP gene was used as an internal reference for gene analyses.

The qRT-PCR analyses were conducted using the Bio-RAD CFX96 Real-time PCR Detection System (Bio-Rad, USA) according to the manufacturer's instructions using the GoTaq qPCR Master Mix (Promega, USA). The PCR reaction system contained 12.5 μl of 2× real-time PCR Master Mix (containing SYBR Green I), 0.5 μl of each primer, 2 μl of the diluted cDNA samples and nuclease-free water in a final volume of 25 μl. The PCR thermal cycling conditions were as follows: 95°C for 3 min, followed by 44 cycles of 95°C for 15 s and 57°C for 35 s, and a final melt curve at 60°C for 5 s to 95°C at 0.5°C increments. Each treatment was technically and biologically conducted independently and in triplicate. The relative gene expression was calculated according to the delta Ct method of the system. To determine gene expressions and standard deviation in different lines, data were statistically analyzed by univariate ANOVO from IBM SPSS Statistics 19.

## Conclusion

With the recent advancement of genome sequencing technology, it has become possible to identify and investigate expression of complex gene families in non-model systems. In this study, we identified 85 putative P450s in *P. xylostella*. By analyzing evolutionary relationships with other insects and the expression patterns of the *Px*P450s, we provided detailed information on their diversity and evolution. The Lepidoptera- specific clusters of CYP367s, which show abundant expression in adult heads, and CYP340s, that are associated with transposable element density, are excellent candidates for investigating detoxification of insecticides and plant toxins. Genomic characterization and gene expression analysis of the *Px*P450s during development will facilitate future functional studies of this complex gene family in *P. xylostella*.

## Supplementary Material

Supplementary Information Supplementary Information

## Figures and Tables

**Table 1:** Family-based numbers of the cytochrome P450 genes and corresponding transposable elements (TEs) identified from the *P. xylostella* genome

P450 clan	Family	Subfamily	No. of genes	Total No. of TEs	Average No. of TEs per gene
CYP4	CYP340	AF, S, T, U, V, W, X, Y, Z	16	1282	80.1
	CYP4	AU, DK, G, M, S	8	350	43.8
	CYP367	A, B	8	401	50.1
	CYP366	C	2	97	48.5
	CYP341	A, G	2	79	39.5
CYP3	CYP6	AB, AE, AN, BD, BF, BG, CN, CT, CV, FM	14	655	46.8
	CYP9	G	5	271	54.2
	CYP365	A	3	128	42.7
	CYP354	A	1	28	28.0
	CYP321	E	1	43	43.0
	CYP429	A	1	30	30.0
	CYP338	C	1	39	39.0
CYP2	CYP304	F	4	244	61.0
	CYP18	A, B	2	28	14.0
	CYP15	C	1	25	25.0
	CYP303	A	1	59	59.0
	CYP307	A	1	58	58.0
	CYP305	B	1	49	49.0
Mitochondrial	CYP333	A, B	3	59	19.7
	CYP301	A, B	2	124	62.0
	CYP428	A	2	128	64.0
	CYP302	A	2	74	37.0
	CYP314	A	1	52	52.0
	CYP49	A	1	25	25.0
	CYP339	A	1	36	36.0
	CYP315	A	1	38	38.0
Total	26	52	85	4402	52.0
