# Supplementary material for: Characterization and expression of the cytochrome P450 gene family in diamondback moth, Plutella xylostella (L.)
Source: Sci Rep. 2015 Mar 10;5:8952. doi: 10.1038/srep08952 (PMC5155450; doi:10.1038/srep08952)
Supplement: Supplementary Information [file srep08952-s1.pdf]

Supplementary Information

Characterization and expression of the cytochrome P450 gene family in diamondback moth, *Plutella xylostella* (L.)

Liying Yu[1,2,3], Weiqi Tang[1,2] , Weiyi He[1,3], Xiaoli Ma[1,3], Liette Vasseur[1,4], Simon W Baxter[5], Guang Yang[1,3], Shiguo Huang[1,3], Fengqin Song[1,2,3], Minsheng You[1, 3]

[1] Institute of Applied Ecology, Fujian Agriculture and Forestry University, Fuzhou 350002, China.

[2] Faculty of Life Sciences, Fujian Agriculture and Forestry University, Fuzhou 350002, China.

[3] Key Laboratory of Integrated Pest Management for Fujian-Taiwan Crops, Ministry of Agriculture, Fuzhou 350002, China.

[4]Department of Biological Sciences, Brock University, St. Catharines, Ontario, Canada.

[5]School of Molecular & Biomedical Science, the University of Adelaide, Adelaide, South Australia, Australia.

This file includes:

Table S1~S2

Figures S1~S5

Table S1 Cytochrome P450 genes in *P. xylostella*.

No.	Gene CDS ID	Cytochrome P450 name	Protein length	Scaffold location	Gene start site	Gene end site	Gene length	No. of exon
**1**	Px000631.1	CYP341G1	448	scaffold_106	535778	541121	5344	8
**2**	Px000833.1	CYP367A4	556	scaffold_109	1288791	1297112	8322	10
**3**	Px000834.1	CYP367B5	500	scaffold_109	1303983	1311031	7049	10
**4**	Px000924.2	CYP340V1v1	494	scaffold_11	200786	212000	11215	10
**5**	Px000926.1	CYP340V2v1	424	scaffold_11	223579	232025	8477	8
**6**	Px001368.1	CYP367B5v2	215	scaffold_1179	13947	16730	2784	4
**7**	Px002079.1<br>Px002080.1	CYP340AF1	458	scaffold_130	925350	930821	5472	9
**8**	Px002082.2b	CYP340T1	503	scaffold_130	950638	958947	8310	10
**9**	Px002082.2a	CYP340S1v1	493	scaffold_130	961179	978354	17176	10
**10**	Px002083.1	CYP340Y1	479	scaffold_130	1000916	1010810	9895	10
**11**	Px002092.2	CYP4S10	507	scaffold_130	1235035	1247351	12317	10
**12**	Px002515.1	CYP305B1	488	scaffold_14	52466	59966	7501	8
**13**	Px002723.1	CYP6CV2	505	scaffold_140	953589	955357	1769	2
**14**	Px002776.1b	CYP340V1v2	346	scaffold_141	688428	693615	5188	7
**15**	Px002776.1a	CYP340V2v2	403	scaffold_141	697194	704079	7538	7
**16**	Px002858.1	CYP304F9v2	363	scaffold_144	157394	161092	3699	6
**17**	Px003086.1	CYP6BD11	527	scaffold_148	1607990	1626691	18702	6
**18**	Px003246.1	CYP6AE45	520	scaffold_15	2271373	2275194	3822	2
**19**	Px003485.1	CYP9G11v1	466	scaffold_154	219752	226769	7018	10
**20**	Px003987.1	CYP367A5v2	162	scaffold_1625	416	2553	2138	3
**21**	Px004291.1	CYP302A1v1	515	scaffold_17	1656307	1660850	4544	9
**22**	Px004643.1	CYP6AB28	512	scaffold_18	104121	106032	1912	2
**23**	Px004650.2	CYP367A5	534	scaffold_18	201458	209934	8477	10
**24**	Px004651.2	CYP367B4v2	484	scaffold_18	216878	225966	9089	12
**25**	Px004867.2	CYP340W1v2	426	scaffold_185	426584	441472	14889	8
**26**	Px004951.2	CYP4DK1v1	505	scaffold_187	507838	516624	8787	11
**27**	Px005514.4	CYP366C1	508	scaffold_20	363581	372702	9122	10
**28**	Px005515.2	CYP366C2	525	scaffold_20	377563	387967	10405	11
**29**	Px005621.2	CYP4DK1v2	452	scaffold_201	206890	210613	3724	10
**30**	Px005900.1<br>Px005901.1	CYP6BG6	524	scaffold_21	1278550	1281643	3094	3
**31**	Px005902.4	CYP6BG4	519	scaffold_21	1287549	1289300	1752	2
**32**	Px006053.5b	CYP4M22	503	scaffold_214	48369	56675	8307	8
**33**	Px006053.5a	CYP4M23	508	scaffold_214	57398	61660	4263	9
**34**	Px006271.1	CYP367B4	500	scaffold_220	512431	519173	6743	10
**35**	Px006272.1	CYP367A5v3	162	scaffold_220	529897	531867	1971	4
**36**	Px006839.1	CYP340X1	466	scaffold_240	366112	376657	10546	10
**37**	Px007099.1	CYP4G78	563	scaffold_25	1012110	1018813	6704	10

No.	Gene CDS ID	Cytochrome P450 name	Protein length	Scaffold location	Gene start site	Gene end site	Gene length	No. of exon
**38**	Px007213.1	CYP340U1v1	559	scaffold_254	6413	12905	8305	12
**39**	Px007214.1	CYP340U2	298	scaffold_254	26018	34339	8322	6
**40**	Px007338.1	CYP333B15	503	scaffold_26	560386	564771	4386	9
**41**	Px007339.2	CYP333B16	502	scaffold_26	566773	570392	3620	9
**42**	Px007344.4	CYP333A5	508	scaffold_26	724089	727671	3583	9
**43**	Px007583.1	CYP307A1	539	scaffold_27	494517	502133	7617	2
**44**	Px008026.1	CYP4G77	560	scaffold_286	50304	54942	4639	7
**45**	Px008489.1	CYP6CN1	496	scaffold_30	1210747	1217884	7138	3
**46**	Px008532.1	CYP340U1v2	415	scaffold_30	2113381	2124856	11476	9
**47**	Px008537.1	CYP340W1v1	495	scaffold_30	2251972	2261032	9061	9
**48**	Px009062.1	CYP304F-1	146	scaffold_32	145580	151208	5630	3
**49**	Px009291.1<br>Px009292.1<br>Px009293.1	CYP304F8	478	scaffold_329	282849	296335	13487	8
**50**	Px009407.1	CYP428A1v2	549	scaffold_331	315475	322803	7329	11
**51**	Px009572.1	CYP338C1	472	scaffold_34	928224	931458	3235	2
**52**	Px009934.1	CYP365A2	456	scaffold_358	28588	34920	6333	8
**53**	Px009935.1	CYP365A5	169	scaffold_358	44166	52002	7837	4
**54**	Px010671.1	CYP339A3	493	scaffold_393	121828	127053	5226	10
**55**	Px010920.1	CYP340Z1	456	scaffold_403	93788	106621	12834	11
**56**	Px011058.5	CYP314A1	525	scaffold_410	156902	168437	11536	9
**57**	Px011481.1	CYP15C1	441	scaffold_436	180461	185272	4812	8
**58**	Px011483.1	CYP429A1	437	scaffold_436	191369	194940	3572	8
**59**	Px011722.1	CYP301A1	519	scaffold_45	130150	145519	15370	8
**60**	Px011724.2	CYP301B1	550	scaffold_45	156061	170376	14316	12
**61**	Px011849.1	CYP302A1v2	476	scaffold_455	200185	204370	4186	8
**62**	Px012360.1	CYP304F-2	153	scaffold_484	199097	201835	2739	3
**63**	Px012411.1	CYP18B1	565	scaffold_49	30802	37118	6317	7
**64**	Px012412.2	CYP18A1	522	scaffold_49	60494	63210	2717	7
**65**	Px012658.1	CYP49A1	503	scaffold_50	610535	617485	6951	8
**66**	Px013017.1	CYP6CT3	518	scaffold_52	1254475	1256852	2378	5
**67**	Px013063.1	CYP340S1v2	418	scaffold_522	117690	128766	11077	8
**68**	Px013091.1	CYP428A1	469	scaffold_526	48482	58721	10240	9
**69**	Px013145.1	CYP6BF1	514	scaffold_53	266651	268456	1806	2
**70**	Px013454.1	CYP6FM1	549	scaffold_552	47611	49492	1882	2
**71**	Px013707.2	CYP341A12	509	scaffold_579	90391	101147	10757	10
**72**	Px013884.1	CYP9G11v2	517	scaffold_59	1385465	1396016	10552	10
**73**	Px013958.1	CYP321E1	500	scaffold_6	239033	241012	1980	2
**74**	Px014069.1	CYP315A1	461	scaffold_600	90089	99740	9652	7
**75**	Px014216.1	CYP6BG5	521	scaffold_613	47293	49077	1785	2
**76**	Px014217.1	CYP6BG3	523	scaffold_613	58716	60633	1918	2
**77**	Px014440.2	CYP354A8	517	scaffold_629	18899	24482	5584	10

No.	Gene CDS ID	Cytochrome P450 name	Protein length	Scaffold location	Gene start site	Gene end site	Gene length	No. of exon
**78**	Px014651.1	CYP6AN14	514	scaffold_65	303284	306224	2941	2
**79**	Px014978.1	CYP9G10	520	scaffold_68	366776	371019	4244	9
**80**	Px014981.1	CYP9G4	519	scaffold_68	524542	532510	7969	10
**81**	Px014982.1<br>Px014984.1	CYP9G2	521	scaffold_68	534795	541015	6221	10
**82**	Px015762.1	CYP4AU6	474	scaffold_74	272693	278565	5873	10
**83**	Px015922.1	CYP303A1	500	scaffold_75	703659	709650	5992	3
**84**	Px016740.1	CYP365A3P	527	scaffold_83	203727	211127	7401	10
**85**	Px017253.1	CYP6AE46	516	scaffold_9	1531564	1533352	1789	2

Table S2 Primers used for qRT-PCR.

No.	P450s	Sense primer	Anti-sense primer	Sense primer Tm (℃)	Anti-sense primer Tm (℃)	Product length (bps)
**1**	CYP307A1	CTCGTGGTCAATAGTTTGGATAGGA	GAAGAGTTGGTGGAACCGCAT	61.8	61.4	99
**2**	CYP314A1	CCAATGAAGGAAGGGTTTAGGC	TGACGATGAGGAAGAGGAGGG	62	61.3	128
**3**	CYP315A1	CAGGTGCTGTTGGCTAAGTTGC	CGTCAGGTGCGTACATGAAGGT	62.5	62.4	94
**4**	CYP18A1	GCGAGGTCCACGAGTTGATTG	GACATGGTGATGCCACAGATGAC	62.7	62.2	112
**5**	CYP4G77	GTCTCGCTTTCTTGGACCTGC	GGTGTCGTGACCCTCGAACAT	61.4	61.8	110
**6**	CYP4G78	CGCAGAACGGAACTCACAACAT	CCAAGGAGGCACAGCACGA	62.5	62.5	118
**7**	CYP301A1	GCGGCTAGGATGCCTGTCTT	GCCAGTAGGGTGCCTTCAGTTC	61.8	62.7	119
**8**	CYP6BF1	CATAACGGGAGACAGCATTGACA	TGGAAGCCAGCTTGGAAGAAC	62.5	61.8	117
**9**	CYP6BG4	CGACAGAACCTCACGCCACT	TCCTCACTTCTACTGCGTCACTCA	60.7	61.7	133
**10**	CYP6BG3	TTTGAAGTGTCGGTATTAAGAGGCT	GAAGAACGCAGGTATTTGTGGG	61.2	61.3	111
**11**	CYP4M22	CAAGAGTTGCGTGAGATATTCGG	GAAGCGTCTCCTTGATACAGCA	61.7	59.7	100
**12**	CYP4M23	CTTCGCTTGTTTCCTCCTGTGT	GCATACTCGCCCATTTCCTCA	61.1	62.4	100
**13**	CYP6BD11	ACCTCCGCCGCTTTCACTCT	CGCACTGGTCCACAAACTTCAT	63.3	62.3	142
**14**	CYP305B1	GATAAGCCTAATGACTTCAACCCAG	TGATTTCGCTAACGCATCTCCT	60.9	61.7	126
**15**	CYP6CN1	AGGATTTCTTCTACGGCATGGTTC	TTGTCTCCCTCGTGCTTCTTCA	63	62.3	139
**16**	CYP9G10	AGAAGGAGGGAAACCAGTGAAA	CAGGCTCTGGGTAATACTGTTCG	59.7	60.9	95
**17**	CYP9G4	GCTGTCCGCATTCATACCTTTC	CCGCGTCTTCTCGCACTTC	61.4	61.5	133
**18**	CYP9G11v1	CGTGAGCGAGACCATGCAGTA	CTTTCTTAGCCTCCATAAGCAGTTGT	62	62.3	83
**19**	CYP6CV2	ATGACAAGGTGGCGAAACTGG	GCTCGTGGATGAGGAAGGACA	62.2	62.2	117
**20**	CYP301B1	GAGTATTTCACCTCTTCCTCCCAGTT	CACATTCGCTTCCCGTATCCA	62.9	63.1	113

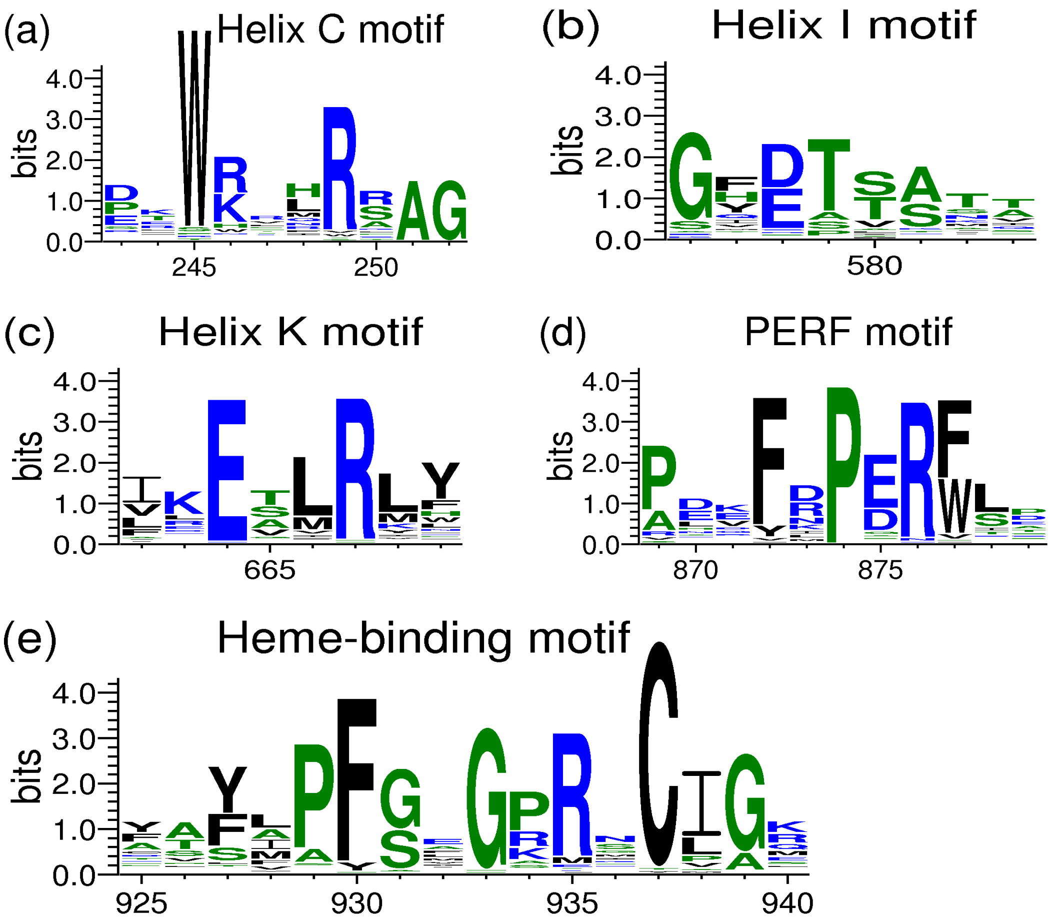

Figure S1 Five conserved domains of the cytochrome P450s in *P. xylostella*. The horizontal axis is the position of amino acids over the entire length of the alignment (1021); the vertical axis represents the information content in bits. Seventy-seven *Px*P450 protein sequences were aligned by ClustalX2, and their conserved motif logos were generated using the online WebLogo program. (a) The helix C motif (WxxxR) sequence logo. (b) The helix I motif (Gx[ED]T[TS]) sequence logo. (c) The helix K motif (ExLR) sequence logo. (d) The PERF motif (PxxFxP[ED]R) sequence logo. (e) The heme-binding motif (PFxxGxRxCx[GA]) sequence logo.

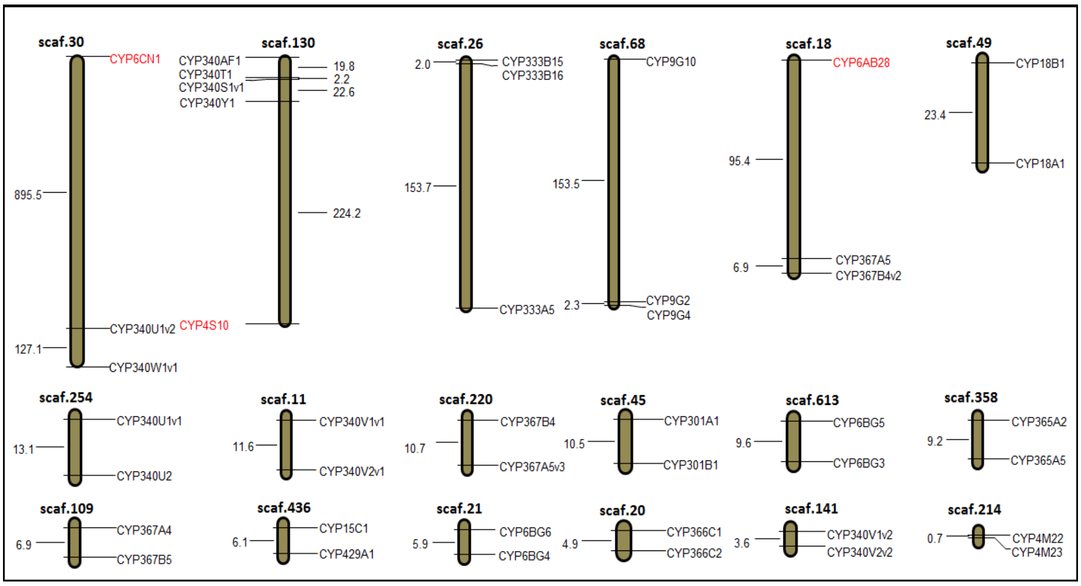

Figure S2 Scaffold localization of cytochrome P450s in *P. xylostella*. The scaffold numbers are indicated at the top of the each bar. The P450 gene names and their distance (kilobases, kb) between two nearby genes are displayed on the right and left of each bar. Tandemly duplicated genes that are supposed to be separated by 0 - 5 genes are in black, and genes that are not tandemly distributed are in red.

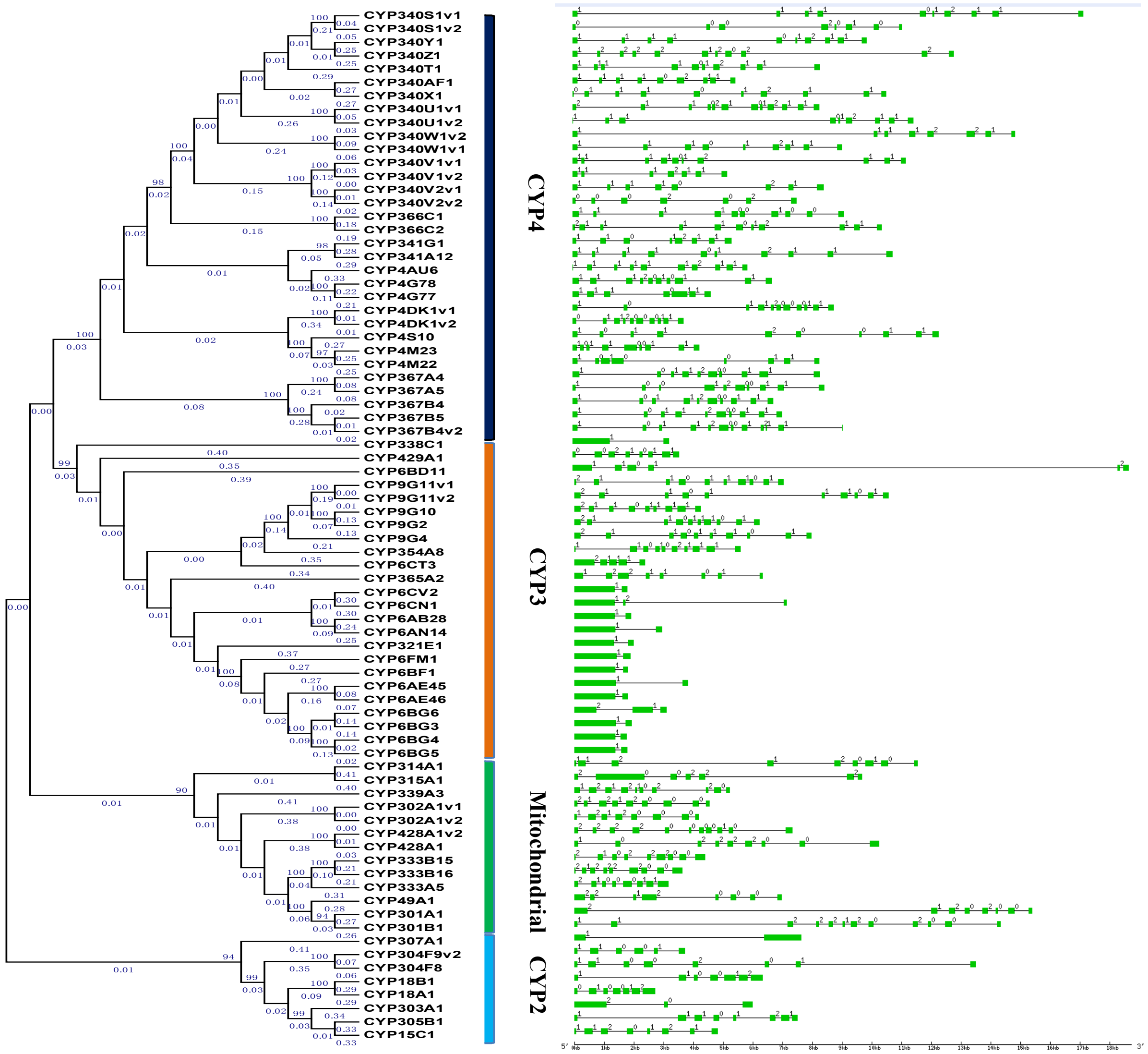

Figure S3 Phylogenetic tree and gene architecture of cytochrome P450s in *P. xylostella*. On the left is the phylogenetic relationship between more than 300 amino acid residues of the P450s. Only Bootstrap values larger than 90% are displayed. On the right is the gene architecture. The green bar represents exons, and black lines represent introns. The digits on the black line represent the intron phase, and three types of intron phases exist in eukaryotic genes and are represented by phases 0, 1, 2. The scaled line on the right bottom measures the gene length.

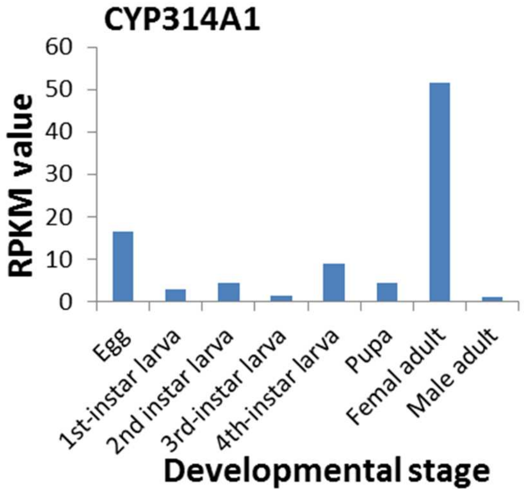
CYP314A1
RPKM value
60
50
40
30
20
10
0
Egg
1st-instar larva
2nd instar larva
3rd-instar larva
4th-instar larva
Pupa
Femal adult
Male adult
Developmental stage

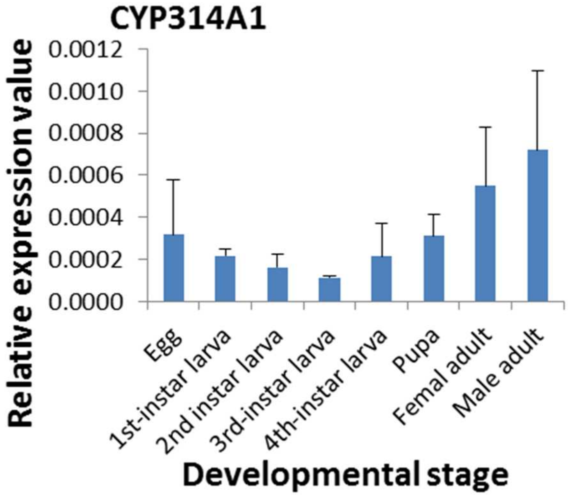
CYP314A1
Relative expression value
0.0012
0.0010
0.0008
0.0006
0.0004
0.0002
0.0000
Egg
1st-instar larva
2nd instar larva
3rd-instar larva
4th-instar larva
Pupa
Femal adult
Male adult
Developmental stage

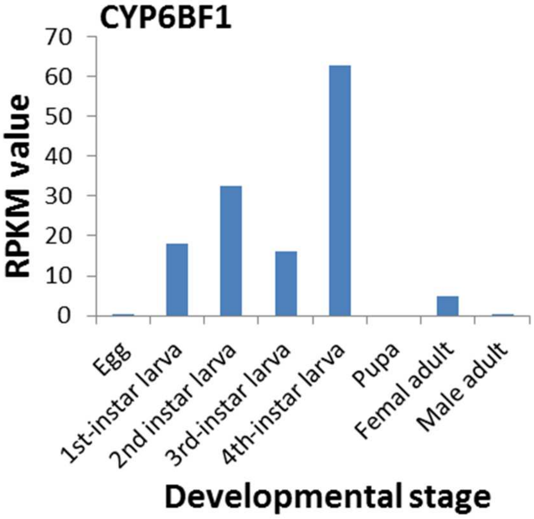
CYP6BF1
RPKM value
70
60
50
40
30
20
10
0
Egg
1st-instar larva
2nd instar larva
3rd-instar larva
4th-instar larva
Pupa
Femal adult
Male adult
Developmental stage

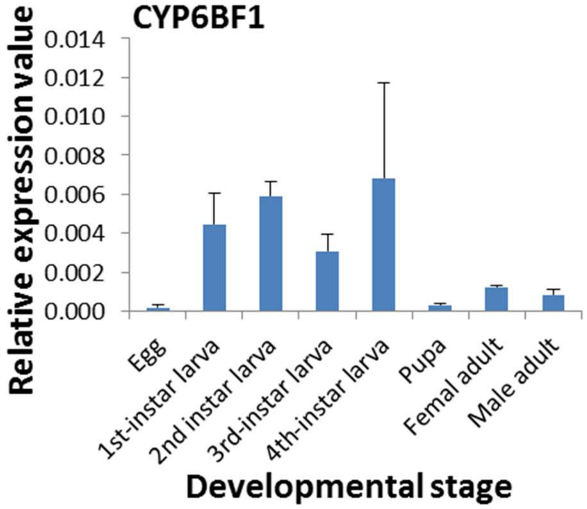
CYP6BF1
Relative expression value
0.014
0.012
0.010
0.008
0.006
0.004
0.002
0.000
Egg
1st-instar larva
2nd instar larva
3rd-instar larva
4th-instar larva
Pupa
Femal adult
Male adult
Developmental stage

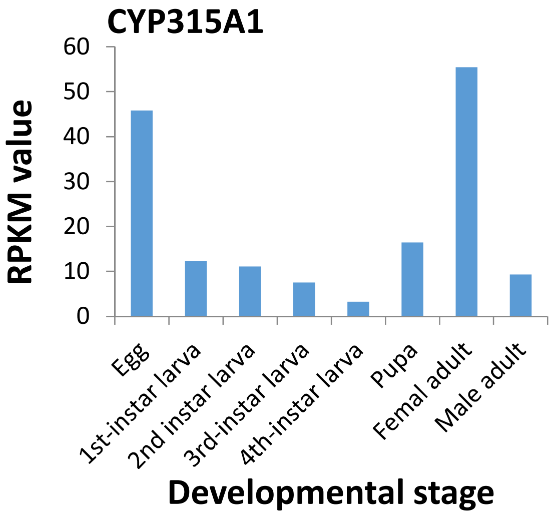
CYP315A1
RPKM value
60
50
40
30
20
10
0
Egg
1st-instar larva
2nd instar larva
3rd-instar larva
4th-instar larva
Pupa
Femal adult
Male adult
Developmental stage

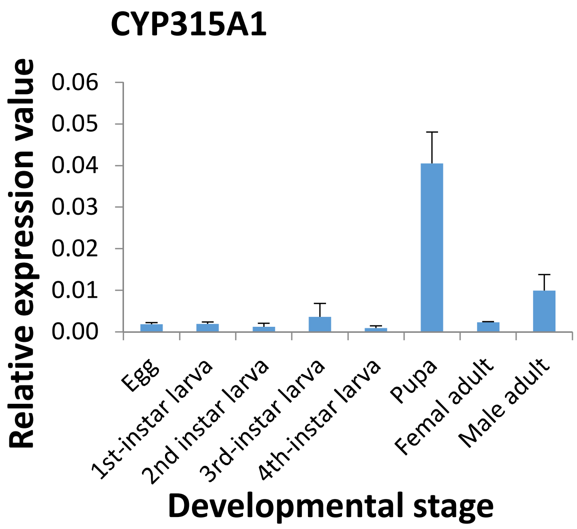
CYP315A1
Relative expression value
0.06
0.05
0.04
0.03
0.02
0.01
0.00
Egg
1st-instar larva
2nd instar larva
3rd-instar larva
4th-instar larva
Pupa
Femal adult
Male adult
Developmental stage

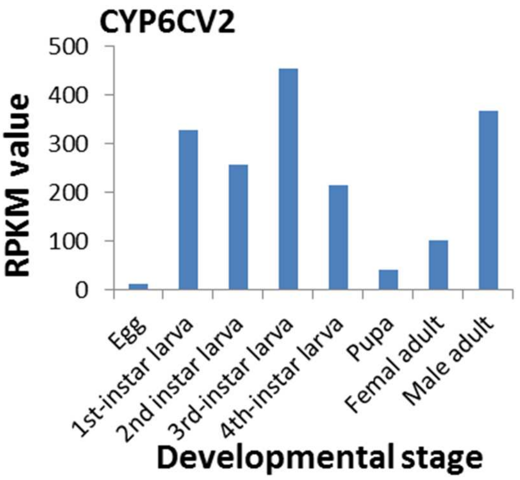
CYP6CV2
RPKM value
500
400
300
200
100
0
Egg
1st-instar larva
2nd instar larva
3rd-instar larva
4th-instar larva
Pupa
Femal adult
Male adult
Developmental stage

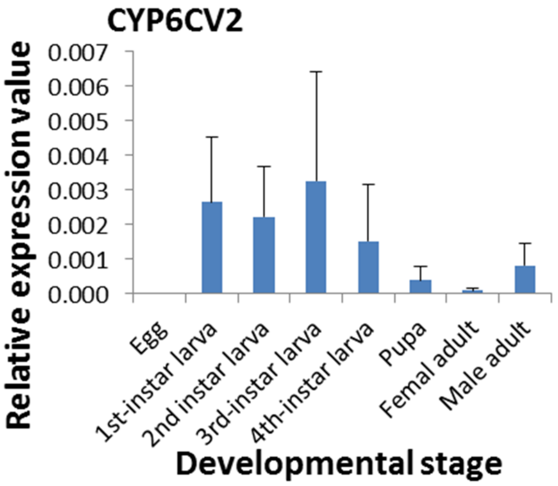
CYP6CV2
Relative expression value
0.007
0.006
0.005
0.004
0.003
0.002
0.001
0.000
Egg
1st-instar larva
2nd instar larva
3rd-instar larva
4th-instar larva
Pupa
Femal adult
Male adult
Developmental stage

73

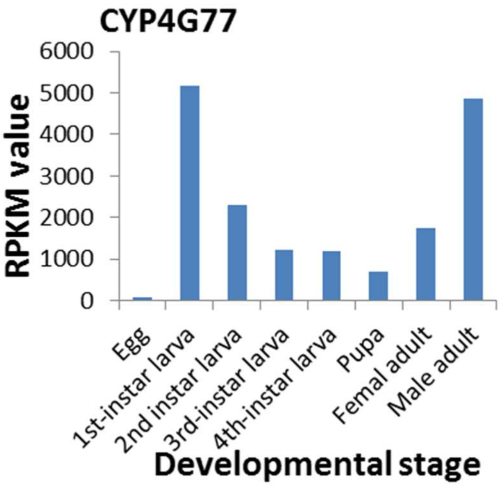
CYP4G77
RPKM value
6000
5000
4000
3000
2000
1000
0
Egg
1st-instar larva
2nd instar larva
3rd-instar larva
4th-instar larva
Pupa
Femal adult
Male adult
Developmental stage

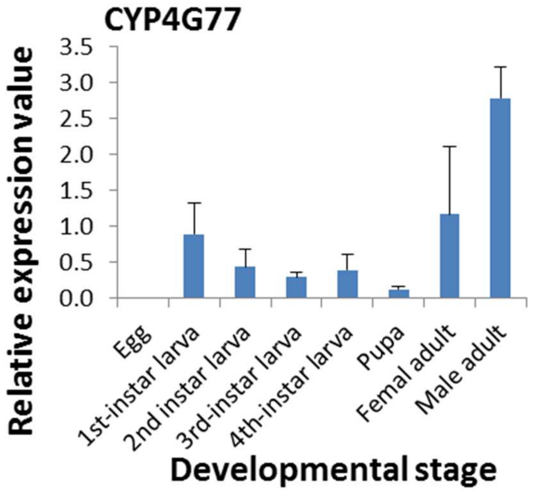
CYP4G77
Relative expression value
3.5
3.0
2.5
2.0
1.5
1.0
0.5
0.0
Egg
1st-instar larva
2nd instar larva
3rd-instar larva
4th-instar larva
Pupa
Femal adult
Male adult
Developmental stage

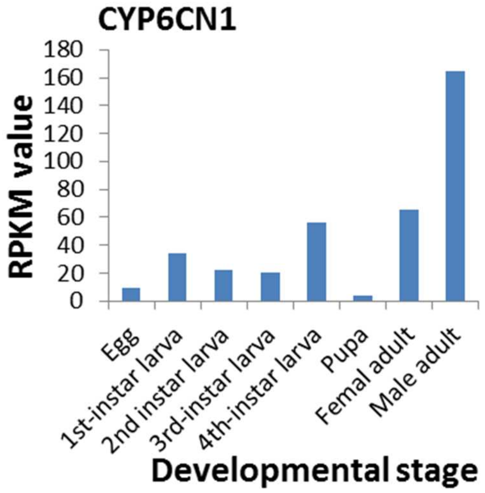
CYP6CN1
RPKM value
180
160
140
120
100
80
60
40
20
0
Egg
1st-instar larva
2nd instar larva
3rd-instar larva
4th-instar larva
Pupa
Femal adult
Male adult
Developmental stage

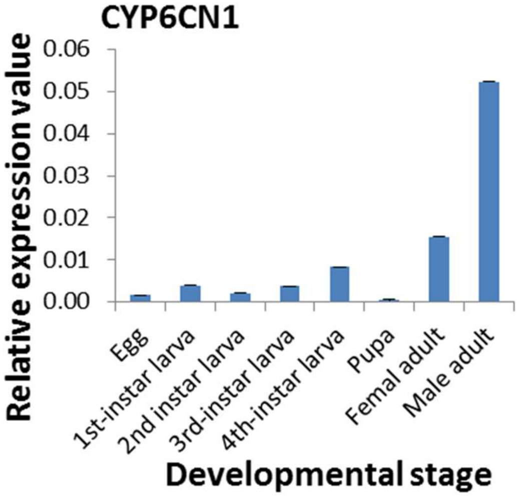
CYP6CN1
Relative expression value
0.06
0.05
0.04
0.03
0.02
0.01
0.00
Egg
1st-instar larva
2nd instar larva
3rd-instar larva
4th-instar larva
Pupa
Femal adult
Male adult
Developmental stage

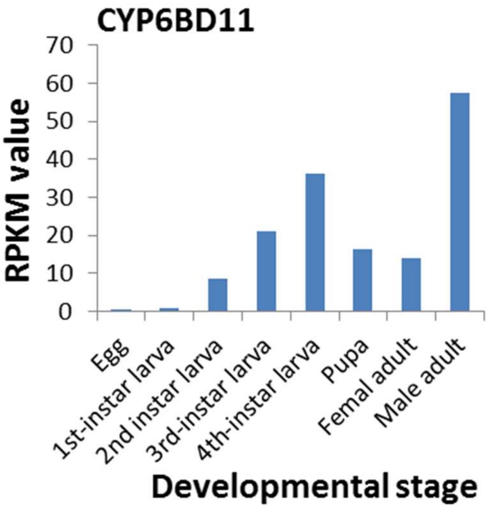
CYP6BD11
RPKM value
70
60
50
40
30
20
10
0
Egg
1st-instar larva
2nd instar larva
3rd-instar larva
4th-instar larva
Pupa
Femal adult
Male adult
Developmental stage

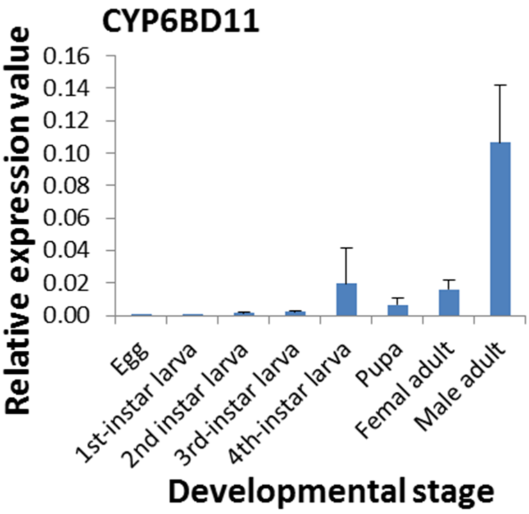
CYP6BD11
Relative expression value
0.16
0.14
0.12
0.10
0.08
0.06
0.04
0.02
0.00
Egg
1st-instar larva
2nd instar larva
3rd-instar larva
4th-instar larva
Pupa
Femal adult
Male adult
Developmental stage

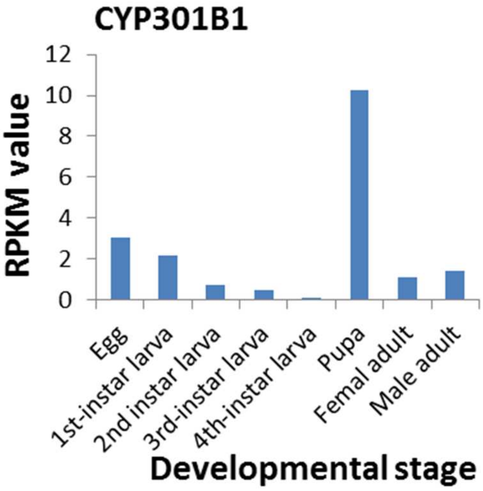
CYP301B1
RPKM value
12
10
8
6
4
2
0
Egg
1st-instar larva
2nd instar larva
3rd-instar larva
4th-instar larva
Pupa
Femal adult
Male adult
Developmental stage

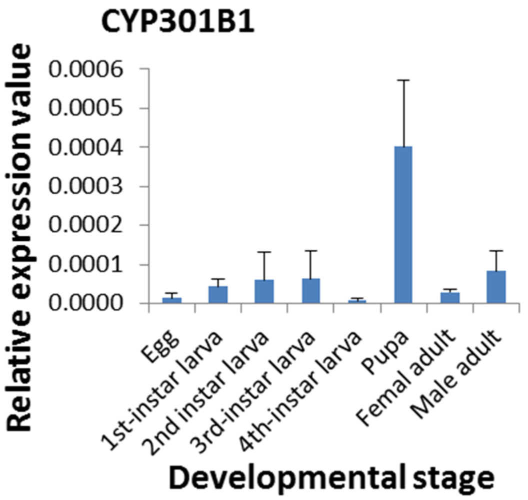
CYP301B1
Relative expression value
0.0006
0.0005
0.0004
0.0003
0.0002
0.0001
0.0000
Egg
1st-instar larva
2nd instar larva
3rd-instar larva
4th-instar larva
Pupa
Femal adult
Male adult
Developmental stage

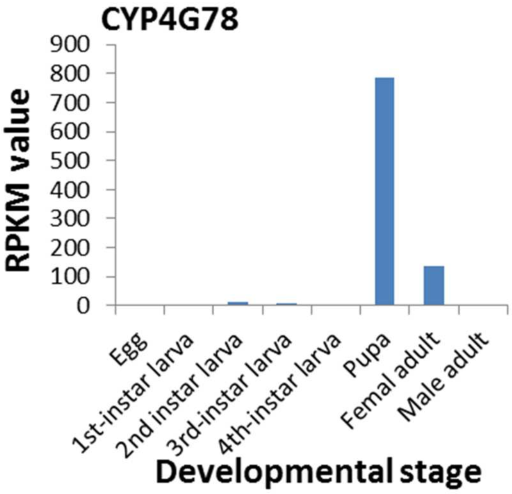
CYP4G78
RPKM value
900
800
700
600
500
400
300
200
100
0
Egg
1st-instar larva
2nd instar larva
3rd-instar larva
4th-instar larva
Pupa
Femal adult
Male adult
Developmental stage

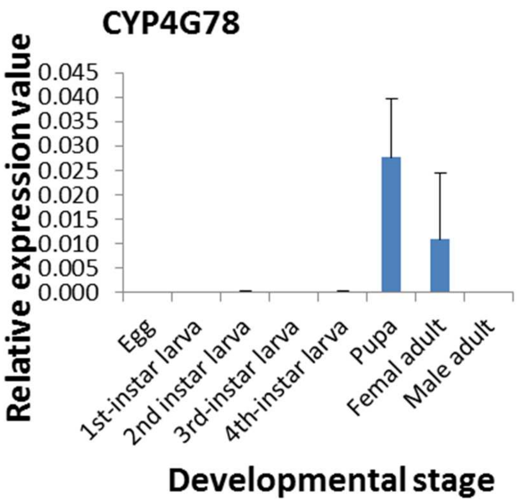
CYP4G78
Relative expression value
0.045
0.040
0.035
0.030
0.025
0.020
0.015
0.010
0.005
0.000
Egg
1st-instar larva
2nd instar larva
3rd-instar larva
4th-instar larva
Pupa
Femal adult
Male adult
Developmental stage

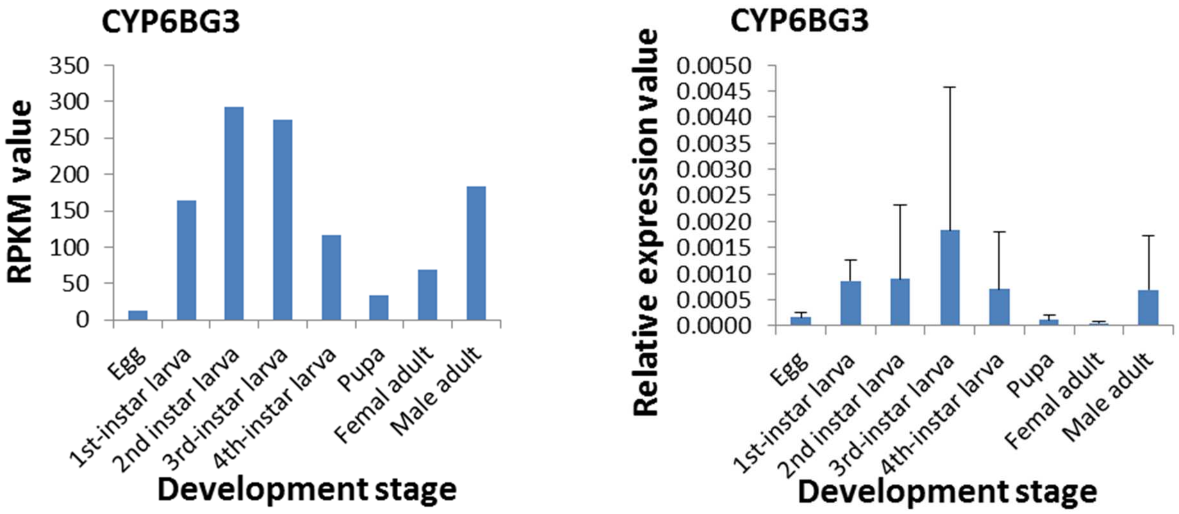

Figure S4 Stage-specific gene expression analysis of 10 *P. sylostella* P450s based on RNA-Seq (left) and qRT-PCR validation (right).

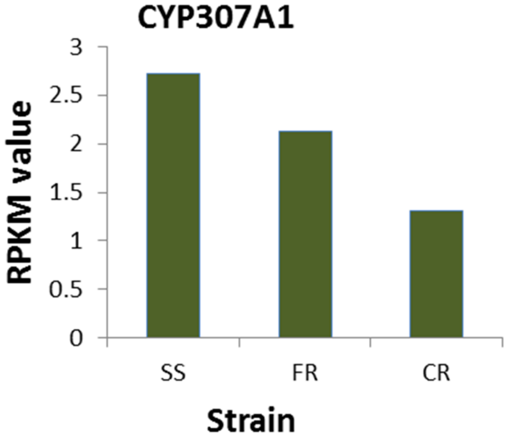
CYP307A1
RPKM value
3
2.5
2
1.5
1
0.5
0
SS
FR
CR
Strain

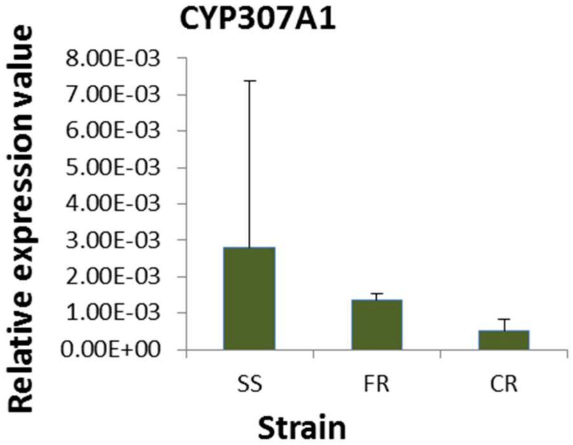
CYP307A1
Relative expression value
8.00E-03
7.00E-03
6.00E-03
5.00E-03
4.00E-03
3.00E-03
2.00E-03
1.00E-03
0.00E+00
SS
FR
CR
Strain

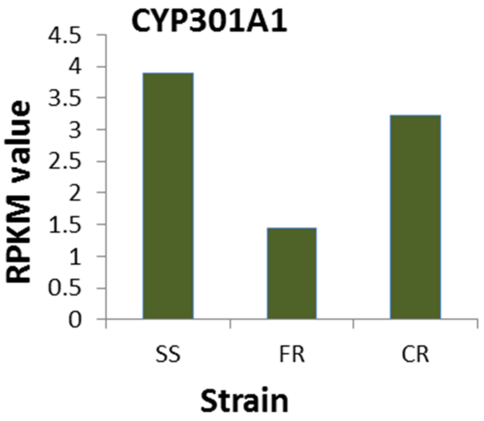
CYP301A1
RPKM value
4.5
4
3.5
3
2.5
2
1.5
1
0.5
0
SS
FR
CR
Strain

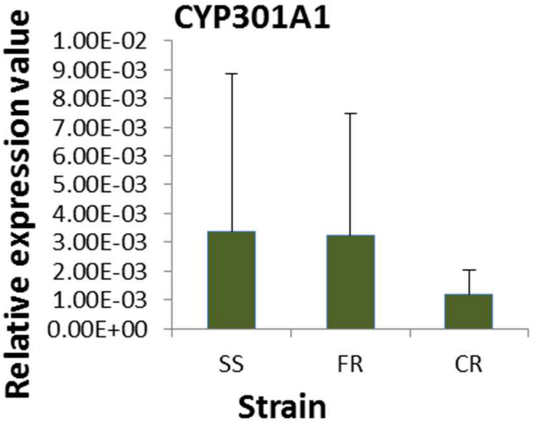
CYP301A1
Relative expression value
1.00E-02
9.00E-03
8.00E-03
7.00E-03
6.00E-03
5.00E-03
4.00E-03
3.00E-03
2.00E-03
1.00E-03
0.00E+00
SS
FR
CR
Strain

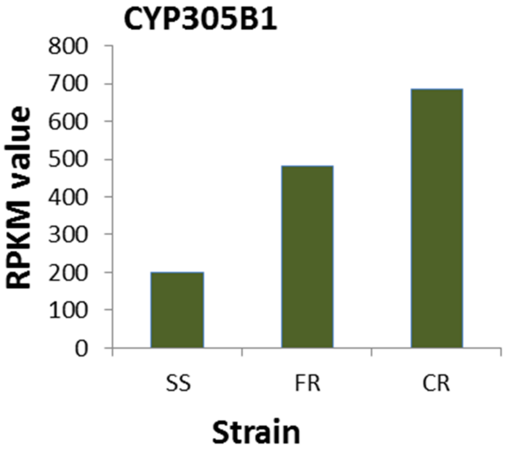
CYP305B1
RPKM value
800
700
600
500
400
300
200
100
0
SS
FR
CR
Strain

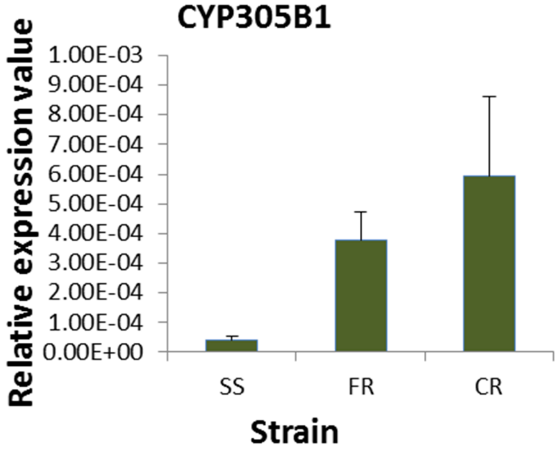
CYP305B1
Relative expression value
1.00E-03
9.00E-04
8.00E-04
7.00E-04
6.00E-04
5.00E-04
4.00E-04
3.00E-04
2.00E-04
1.00E-04
0.00E+00
SS
FR
CR
Strain

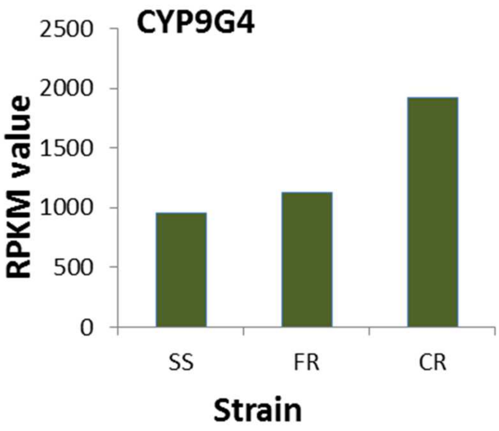
CYP9G4
RPKM value
2500
2000
1500
1000
500
0
SS
FR
CR
Strain

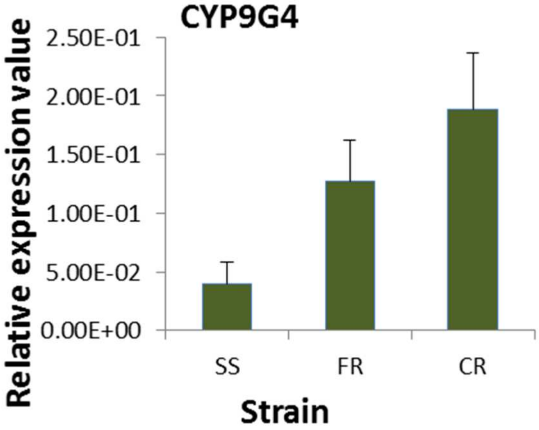
CYP9G4
Relative expression value
2.50E-01
2.00E-01
1.50E-01
1.00E-01
5.00E-02
0.00E+00
SS
FR
CR
Strain

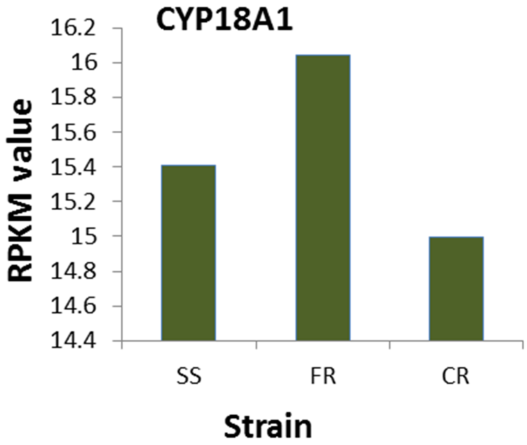
CYP18A1
RPKM value
16.2
16
15.8
15.6
15.4
15.2
15
14.8
14.6
14.4
SS
FR
CR
Strain

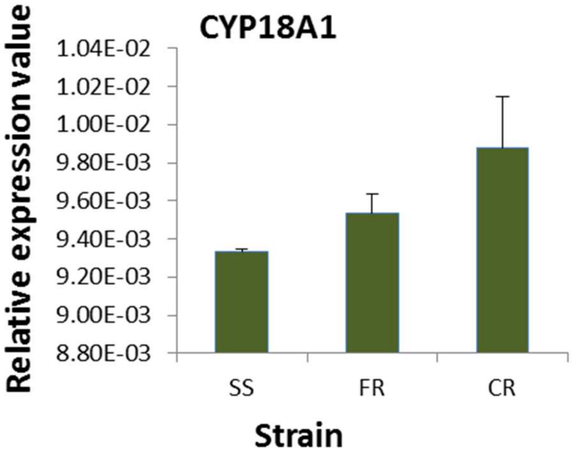
CYP18A1
Relative expression value
1.04E-02
1.02E-02
1.00E-02
9.80E-03
9.60E-03
9.40E-03
9.20E-03
9.00E-03
8.80E-03
SS
FR
CR
Strain

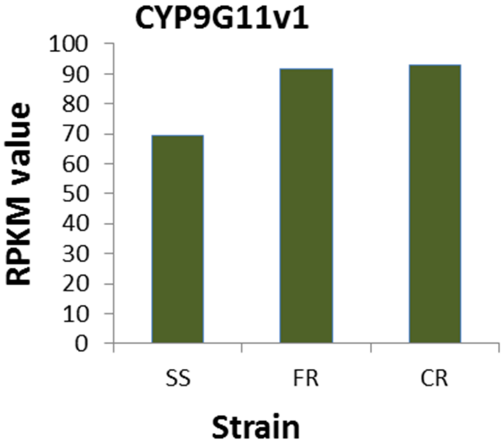
CYP9G11v1
RPKM value
100
90
80
70
60
50
40
30
20
10
0
SS
FR
CR
Strain

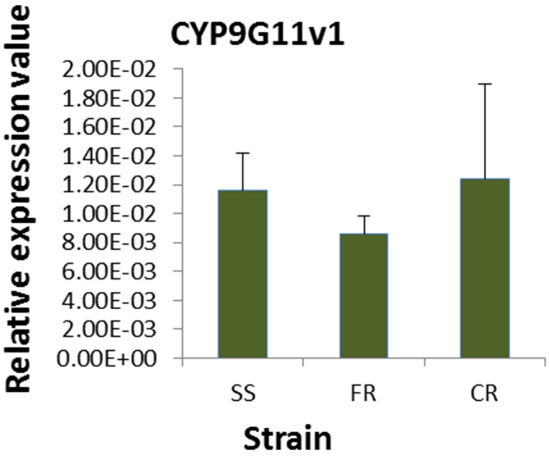
CYP9G11v1
Relative expression value
2.00E-02
1.80E-02
1.60E-02
1.40E-02
1.20E-02
1.00E-02
8.00E-03
6.00E-03
4.00E-03
2.00E-03
0.00E+00
SS
FR
CR
Strain

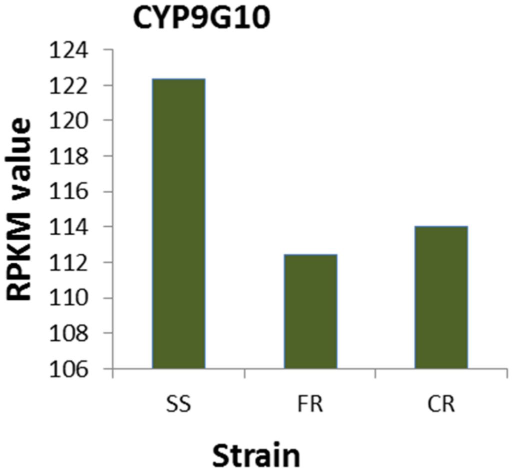
CYP9G10
RPKM value
124
122
120
118
116
114
112
110
108
106
SS
FR
CR
Strain

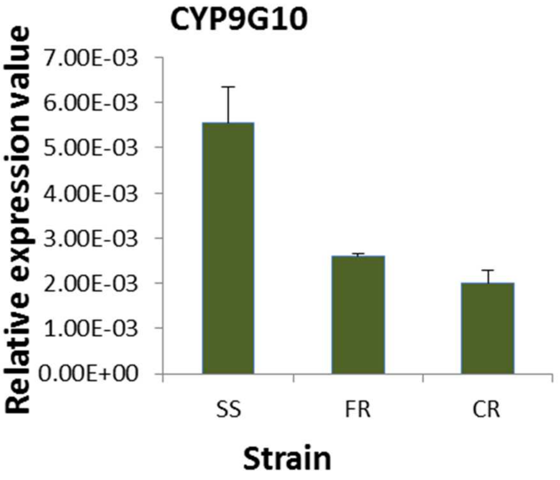
CYP9G10
Relative expression value
7.00E-03
6.00E-03
5.00E-03
4.00E-03
3.00E-03
2.00E-03
1.00E-03
0.00E+00
SS
FR
CR
Strain

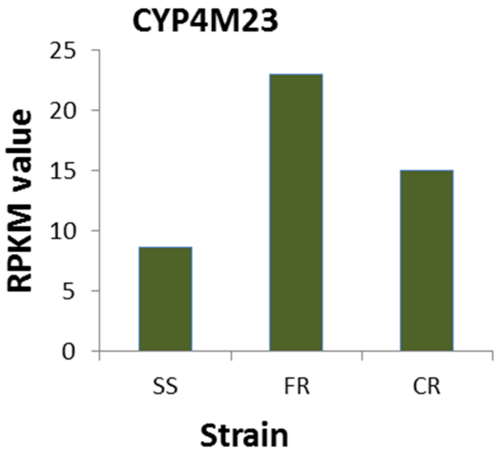
CYP4M23
RPKM value
25
20
15
10
5
0
SS
FR
CR
Strain

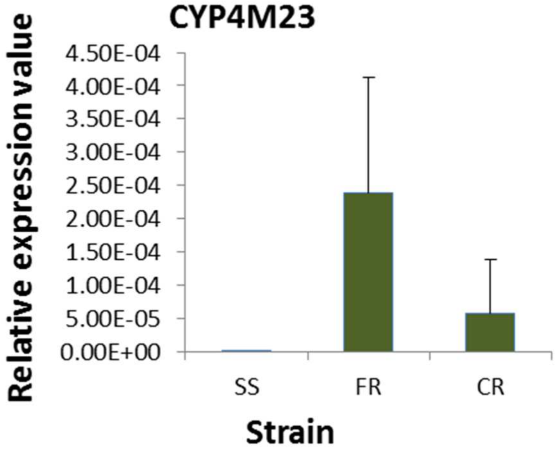
CYP4M23
Relative expression value
4.50E-04
4.00E-04
3.50E-04
3.00E-04
2.50E-04
2.00E-04
1.50E-04
1.00E-04
5.00E-05
0.00E+00
SS
FR
CR
Strain

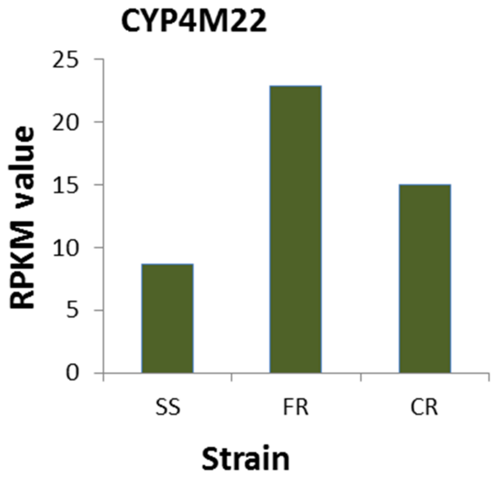
CYP4M22
RPKM value
25
20
15
10
5
0
SS
FR
CR
Strain

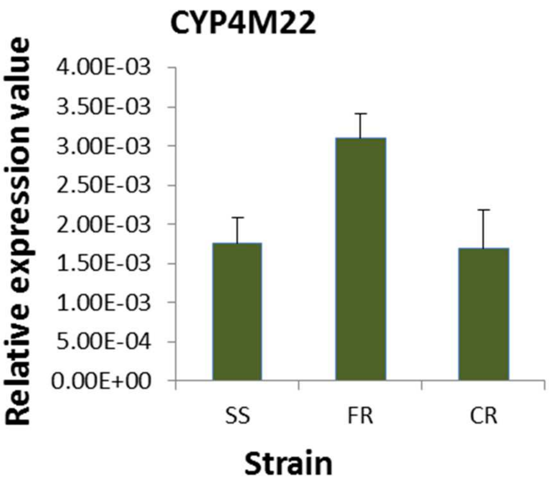
CYP4M22
Relative expression value
4.00E-03
3.50E-03
3.00E-03
2.50E-03
2.00E-03
1.50E-03
1.00E-03
5.00E-04
0.00E+00
SS
FR
CR
Strain

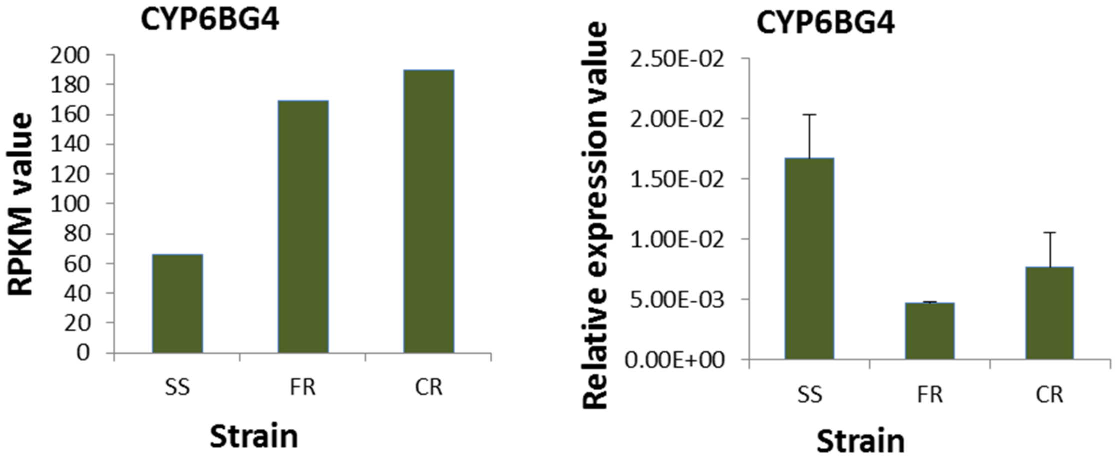

Figure S5 Strain-specific gene expression analysis of 10 *P. xylostella* P450s based on RNA-Seq (left) and qRT-PCR validation (right). SS represents 3rd-instar larva from the susceptible strain; FR 3rd-instar larva from the fipronil-resistant strain; and CR 3rd-instar larva from the chlorpyrifos-resistant strain.
